# Evaluation of Concrete Carbonation Based on a Fiber Bragg Grating Sensor

**DOI:** 10.3390/mi15010029

**Published:** 2023-12-22

**Authors:** Jianzhi Li, Haiqun Yang, Handong Wu

**Affiliations:** 1 Key Laboratory of Structural Health Monitoring and Control, Shijiazhuang Tiedao University, Shijiazhuang 050043, China; 2 School of Materials Science and Engineering, Shijiazhuang Tiedao University, Shijiazhuang 050043, China; yanghaiqun947@163.com (H.Y.); znclsys208@126.com (H.W.)

**Keywords:** concrete carbonation, fiber Bragg grating sensor, carbonation depth, compressive strength, elastic modulus

## Abstract

The carbonation of concrete greatly affects its service life. In this paper, fiber Bragg grating (FBG) sensors were used to investigate the relationship between concrete carbonation and its mechanical properties. A T130 High Sensitivity Strain Cable Sensor with a good linearity was used to monitor the internal strain in concrete, to investigate the variation in the elastic modulus of concrete with carbonation time. A mathematical model of elastic modulus and carbonation time of concrete based on FBG was established. At the same time, the authors explored the relationship between the carbonation depth and compressive strength of concrete and the carbonation time using a phenolphthalein solution test and a compressive strength test, respectively. The experimental results indicate that the carbonation depth, compressive strength, and elastic modulus of concrete increase with carbonation time. In the early stage of carbonation, these three parameters increase rapidly, while they grow slowly in the later stage of carbonation. The varying trend of the elastic modulus of concrete is consistent with the compressive strength, which shows a binomial relationship. Therefore, the elastic modulus, measured using FBG sensors, is used as an indicator of the characterization of the carbonation resistance of concrete. This work provides a new approach for concrete carbonation detection and assessment.

## 1. Introduction

The carbonation and corrosion of reinforcement bars in concrete are significant factors that shorten the service life of concrete structures [1]. Carbonation affects reinforced concrete by neutralizing the concrete and causing the protective layer to fail. When the pH level is above 11.5, a dense passivation film structure forms around the steel bar to resist corrosion. CO_2_ in the air penetrates into the interior of the concrete through the pores and microcracks of the concrete and generates carbonate and water after a chemical reaction with the alkaline substances therein [2,3], resulting in a decrease in the alkalinity of the concrete [4,5]. When the pH level drops below 9.8 and the carbonation depth exceeds the thickness of the concrete cover, the passivation film on the surface of the reinforcement will fail, leading to steel bar corrosion [6,7,8,9]. The expansion in volume resulting from the formation of corrosion products will induce further propagation of cracks, thereby accelerating the process of corrosion and initiating a vicious cycle of deterioration in the concrete structure, which includes the detachment of the protective layer, a reduction in load-bearing capacity, and a reduction in bonding performance, ultimately limiting the service life of reinforced concrete structures. With the growing demand for energy, industry, and transportation, the emissions of greenhouse gases, such as those from industrial and vehicle exhausts, have caused a rise in CO_2_ levels in the air, which poses a significant threat to concrete structures [10]. Therefore, it is of great significance to evaluate the carbonation of concrete.

Numerous scholars have developed various concrete carbonation prediction models to characterize the degree of carbonation [11]. A widely accepted model is the carbonation empirical model based on Fick’s first law, which posits that the depth of carbonation in concrete is directly proportional to the square root of the carbonation time [12]. To accommodate various external environments and specific concrete mix proportions, researchers have extended the scope of this model. Zhu developed a formula for carbonation rate, which took into account the water–cement ratio of concrete as the primary parameter through accelerated carbonation tests, outdoor exposure experiments, and engineering investigation results [13]. Zou et al. investigated the influence of axial compressive stress on the carbonation resistance of recycled coarse aggregate concrete (RAC) and established a prediction model for the carbonation depth of RAC under axial compressive stress, based on theoretical analysis and experimental results [14]. Pan et al. presented a carbonation model based on the crossed influence of multi-factors (such as stress levels, CO_2_, water–cement ratios, and carbonation ages) by regression fitting and analysis of the test data [15]. However, these models are based on laboratory data and are inadequate in fully depicting the actual carbonation process of concrete in natural environments. As a result, it is crucial to obtain data on concrete carbonation in practical engineering applications.

Conventionally, the measurement of carbonation depth using phenolphthalein solution is the primary method for evaluating the carbonation of concrete [16]. Jones et al. conducted a study on measuring the depth of carbonation using phenolphthalein reagent, by observing the change in pH value before and after concrete carbonation [17]. In addition, Kobayashi et al. used quantitative X-ray diffraction analysis (QXRD) to measure the relative content of Ca(OH)_2_ and CaCO_3_ in concrete at different carbonation depths, to evaluate the carbonation depth of concrete [18]. Furthermore, researchers have investigated the correlation between the mechanical properties of concrete, including elastic modulus, compressive strength, and brittleness, and the degree of carbonation [19,20]. Nevertheless, these methods are currently confined to laboratory settings and are inherently destructive, rendering them unsuitable for large-scale structures and critical components in engineering projects. Recently, non-destructive testing methods have been applied to the study of concrete carbonation, such as non-linear ultrasonic techniques [21], resistivity measurement [22,23], and electrochemical impedance spectroscopy [24], etc. Kim used a non-contact air-coupled transducer to detect Rayleigh wave signals and obtained the nonlinear parameters of the material. This allowed for a quantitative evaluation of the degree of carbonation in concrete [25]. However, the detection results are susceptible to contact conditions and the robustness of the method cannot be effectively guaranteed. Singh et al. measured the resistivity of self-compacting concrete with different recycled concrete aggregates contents by using the four-probe method to investigate the differences in its carbonation resistance performance [26]. Dong et al. established a new electrochemical model based on electrochemical impedance spectroscopy to predict the carbonation depth of concrete [27]. These techniques have the advantage of protecting the concrete structure from being damaged. However, they are susceptible to the cracks and pores in the concrete itself and environmental factors such as electromagnetic waves, temperature, humidity, which makes it costly [28].

In recent years, FBG sensing technology has been widely used in the performance research and damage monitoring of concrete structures [29]. Compared with other non-destructive testing methods, it has the advantages of anti-electromagnetic interference, reusability, corrosion resistance, small size, low cost, and the ability to achieve continuous real-time online monitoring [30]. However, there have been few studies utilizing FBG sensors for the evaluation of concrete carbonation. The purpose of this paper is to investigate the relationship between the elastic modulus of concrete, measured by FBG sensors, and the degree of carbonation. The authors provide a new method for evaluating the carbonation and mechanical degradation of concrete.

## 2. Materials and Tests

### 2.1. Concrete Specimens

C30 concrete is a frequently utilized grade of concrete. Its performance basically satisfies the strength, durability, and seismic requirements of diverse engineering projects, ensuring their safety and reliability. On the other hand, it exhibits good malleability and fluidity, rendering it easy to fabricate and shape, and is also economically viable. Therefore, this material is widely used in engineering fields like building structures, bridge engineering, water conservancy, tunnel engineering, and more. However, it is prone to microcracks and has relatively more pores, making it more susceptible to carbonation. Thus, C30 grade concrete was selected for these experiments.

The mix ratio design refers to the specification for mix proportion design of ordinary concrete (JGJ55-2011) [31], as shown in Table 1. The cement applied in these tests was 42.5 ordinary Portland cement, manufactured by Hebei Jinyu Dingxin Cement Co., Ltd. (Shijiazhuang, China). The fine aggregate was natural river sand with a fineness modulus of 2.7 and a moisture content of 4.2%. The coarse aggregate was a continuous graded limestone crushed stone with a particle size from 5 to 20 mm, and its apparent density was 2750 kg/m^3^. All indexes were compliant with the standard for technical requirements and a test method of sand and crushed stone (or gravel) for ordinary concrete (JGJ52-2006) [32].

The slump of the concrete was measured to be 35 mm, which satisfied the standard for test methods of concrete physical and mechanical properties (GB/T50081-2019) [33].

Three batches of specimens were poured for these tests. In the first batch of specimens, a total of nine specimens with a dimension size of 100 mm × 100 mm × 100 mm were tested for their compressive strength at the curing age of 3 d, 7 d, and 28 d using the GB/T50081-2019 standard [33]; in the second batch of specimens, a total of 24 specimens with a dimension size of 100 mm × 100 mm × 100 mm were used to test the depth of carbonation and compressive strength after carbonation for 3 d, 7 d, 14 d, and 28 d; in the third batch of specimens, a total of 12 specimens with a dimension size of 100 mm × 100 mm × 100 mm embedded with FBG sensors were fabricated, to test their elastic modulus at the carbonation time of 7 d, 14 d, 21 d, and 28 d.

Although bare FBG can function as a strain sensor, its small diameter and fragility make it prone to breakage. This presents challenges when adapting to the harsh construction environment on a construction site, such as mechanical vibrations and the flipping of concrete during the pouring process. Therefore, it is necessary to adopt appropriate packaging methods and installation techniques to resist external impact. 

**Figure 1:**
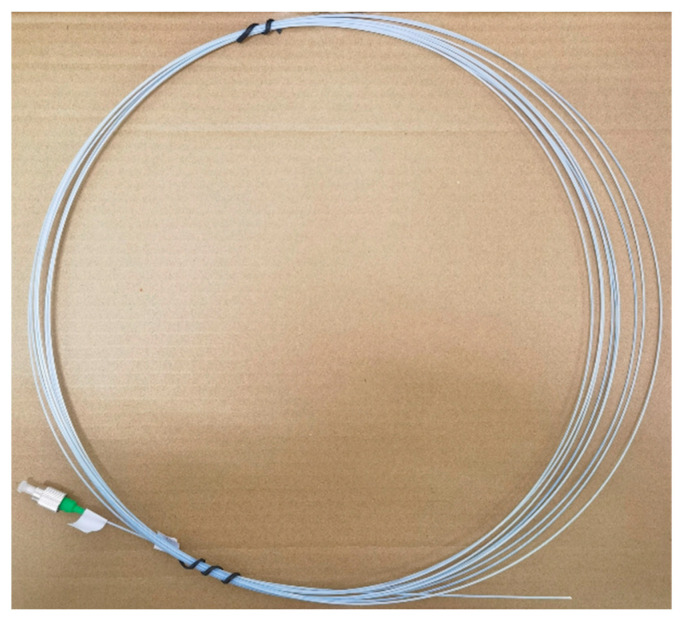
T130 High Sensitivity Strain Cable Sensor.

In this work, a T130 High Sensitivity Strain Cable Sensor was used to monitor the internal strain in concrete (See Figure 1). It consists of a FBG and glass fiber-reinforced plastics (GFRP). The FBG was manufactured on a single-mode silica fiber and the fiber is a standard silica fiber. Thereafter, the FBG was embedded in GFRP and at the center of a GFRP cable. The outer layer of the cable is a GFRP coat, which protects the FBG from damage. GFRP exhibits high strength and corrosion resistance, which makes FBG much more robust in concrete applications. The performance parameters of the T130 are shown in Table 2. 

**Figure 2:**
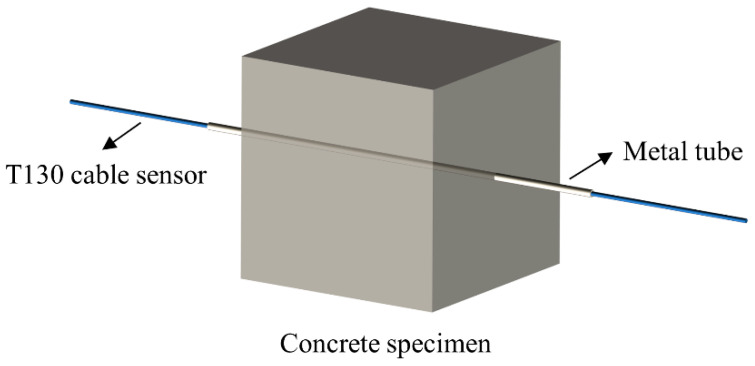
Method of concrete placement with the T130 cable sensor.

Furthermore, the authors used the method of tube insertion to embed T130 into the interior of the concrete, effectively avoiding the external force impact generated during the pouring and compacting of the concrete. Figure 2 shows the method of embedding T130 cable sensors into concrete specimens. The T130 cable sensor was taken and snapped into a metal conduit to prevent it from being damaged. We drilled through the holes reserved in the middle of both sides of the mold used for pouring concrete, ensuring that the sensor was at the center of the concrete specimen, and then the concrete mixture was poured into the mold. Subsequently, the metal conduit was pulled out of the mold after a dense vibrating process. In this way, the T130 cable sensor was effectively bonded to the concrete. The FBG had a more uniform stress distribution at the center of the concrete. Therefore, the authors embedded the FBG in the center of the cubic concrete specimen, to ensure that the strain changes measured by the FBG during the compressive strength test of the concrete specimens at different carbonation times were from the same position.

The compressive strength of the concrete specimens was tested at curing ages of 3 d, 7 d, and 28 d, according to the GB/T50081-2019 standard [33], as shown in Table 3. The specimens that had been poured satisfied the code for design of concrete structures (GB50010-2010) [34] and were suitable for use in following experiments.

### 2.2. Carbonation Depth Test

**Figure 3:**
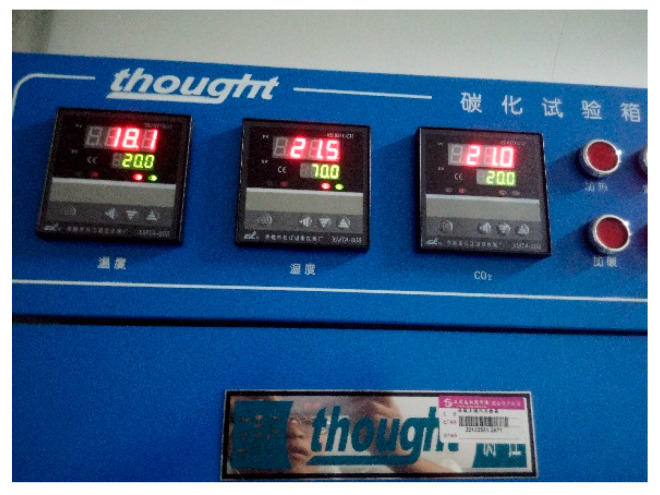
HTH-180 carbonation test chamber.

The concrete specimens were subjected to accelerated carbonation, based on the standard for test methods of long-term performance and durability of ordinary concrete (GB/T50082-2009) [35]. The specimens were removed from the curing room two days prior to testing and allowed to stand at a temperature of 60 °C for 48 h to dry, so as to prevent excessive initial moisture from preventing CO_2_ from entering the specimens and affecting the accuracy of carbonation depth measurement. After drying, the opposite side surfaces of the specimens were reserved, and the rest were sealed by applying epoxy resin. The centerline of the specimens was then drawn with a pencil along the length of the exposed surface, to facilitate the measurement of the carbonization depth. The sealed concrete specimens were positioned in the carbonation test chamber with a minimum distance of 50 mm between them to eliminate the error in carbonation depth resulting from uneven CO_2_ absorption. The carbonation test chamber for the concrete was the HTH-180, produced by Beijing Sansixing Measuring and Controlling Technology Co., Ltd. (Beijing, China) (see Figure 3). The conditions of carbonation are shown in Table 4.

**Figure 4:**
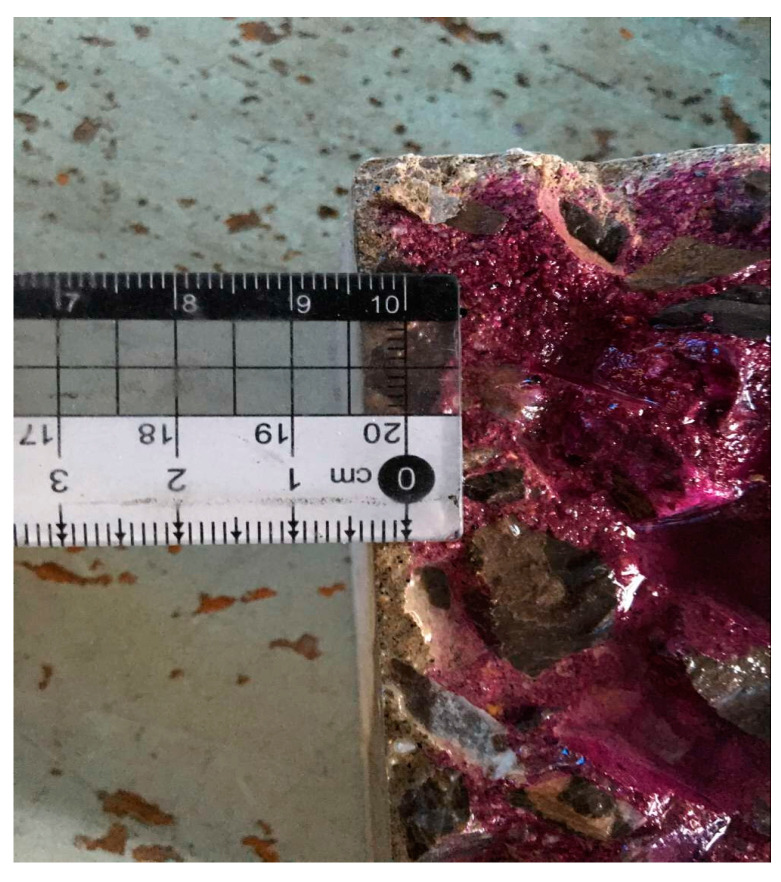
Carbonation depth measurement.

When the carbonation time reached 3 d, 7 d, 14 d, and 28 d, the specimens were taken out and split in the middle by a pressure machine. Subsequently, the authors cleaned up the remaining powder on the split surface and sprayed a solution of phenolphthalein with a concentration of 1%. At least six points were chosen to measure the carbonation depth after the surface became completely discolored and clear (See Figure 4). Following the GB/T50082-2009 standard [35], three tests were conducted at each carbonation time and the arithmetic average of the carbonation depth obtained from these tests was considered to be the measurement value. If there were aggregate particles embedded exactly on the carbonation boundary line at the measuring point, the average value of the carbonation depth on both sides of the particle were taken as the depth value of that point.

### 2.3. Compressive Strength Test after Carbonation

**Figure 5:**
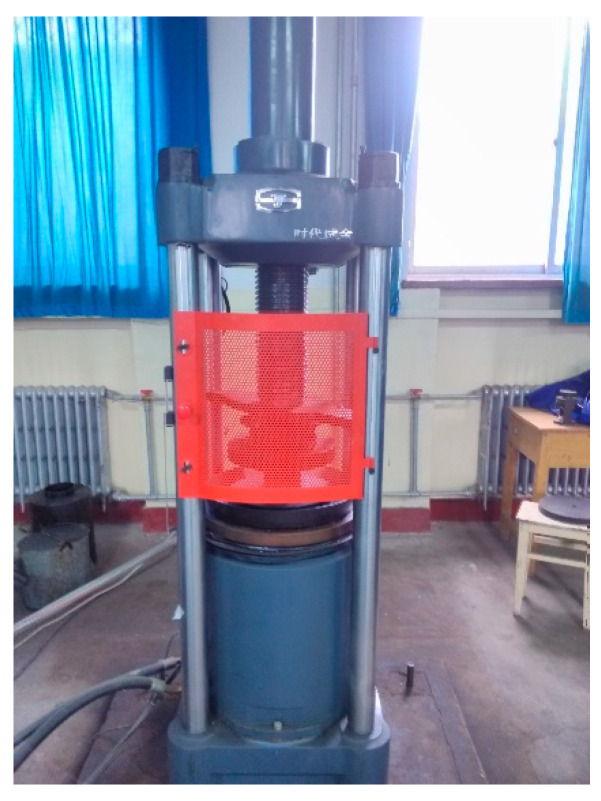
Pressure machine.

The carbonized specimens were tested for compressive strength at the carbonation time of 3 d, 7 d, 14 d, and 28 d. Following the GB/T50081-2019 standard [33], three tests were conducted at each carbonation time and the arithmetic average of compressive strength obtained from these tests was considered to be the test value. The digital display hydraulic pressure machine used is produced by TianShui HongShan Testing Machine Co., Ltd. (Tianshui, China) (See Figure 5). Its range is 0~2000 kN.

### 2.4. Elastic Modulus Test after Carbonation

FBG is a diffraction grating made by a specific method that causes an axial periodic change in the refractive index of the fiber core. In general, when an optical signal passes through the grating area, the optical signal (with a wavelength of $\lambda_{B}$) that satisfies the reflection condition will be reflected back, and the other wavelengths of the optical signal will pass through the FBG, resulting in two types of spectra: reflection and transmission. The wavelength ($\lambda_{B}$) is determined by the effective refractive index ($n_{eff}$) of the fiber core and the refractive index modulation period (Λ), as shown in Equation (1). When the grating is axially stressed, the FBG deforms, i.e., the grating period changes, and the wavelength of the reflected light changes. When an external physical parameter, such as temperature or stress, is applied to the FBG, it causes a shift in the wavelength of the reflected or transmitted spectrum. By detecting this shift, the change in the physical quantity to be measured can be determined. Their relation is shown in Equation (2).

(1) $$\lambda_{B}=2n_{eff}\Lambda$$

(2) $$\frac{∆\lambda_{B}}{\lambda_{B}}=(1-P_{e})∆\varepsilon +(\alpha_{f}+\xi )∆T$$

where $\lambda_{B}$ is the initial wavelength; $∆\lambda_{B}$ is the wavelength shift; $P_{e}$ is the elastic-optic coefficient (approximately 0.22 at ordinary temperature); ∆ε is the strain variation; $\alpha_{f}$ is the thermal expansion coefficient; ξ is the thermo-optic coefficient; and ∆T is the temperature variation.

The wavelength of FBG is directly associated with the load-bearing characteristics of concrete rather than the chemical carbonation. There is an indirect relationship between the carbonation and the wavelength of FBG. Carbonation affects the strength of concrete, subsequently causing varied internal strain upon loading, which in turn affects the FBG wavelength shifts. Therefore, the authors embedded the FBG into the interior of the concrete and measured the elastic modulus of the concrete at different carbonation times by applying a step load, thus establishing the relationship between the elastic modulus and carbonation time.

**Figure 6:**
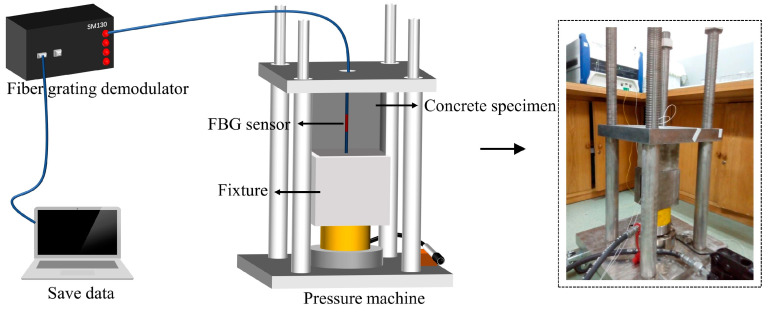
Test of elastic modulus using FBG after carbonation.

The specimen with the FBG sensor was placed on a specially designed pressure machine and the FBG sensor was connected to the SM130 FBG demodulator produced by Micron Optics, Inc. (Atlanta, GA, USA). The load capacity of the specialized press machine is 300 kN. The scan frequency and bandwidth of SM130 are 1000 Hz and 80 nm, respectively. The test diagram is shown in detail in Figure 6. A graded load (10 kN) was applied from 0 kN to 100 kN, ensuring that the ultimate load applied each time remained within the elastic deformation range of the concrete structure. As shown in Figure 6, the concrete specimen generated axial strain under external loads. The strain of concrete was transmitted to the FBG through shear stress, ultimately resulting in a FBG wavelength shift. If the FBG is positioned horizontally, it is insensitive to compressive stress. In this work, the compression test was undertaken in a controlled laboratory environment over a 10 min period. Accordingly, the temperature-induced FBG wavelength shift had no effect on the elastic modulus test in a short test time. Hence, FBG temperature compensation was unnecessary in this experiment.

The FBG wavelength shifts were recorded and saved by SM130 FBG demodulator for every 10 kN applied, and then the strain of the concrete was obtained by the wavelength data. The ratio of stress variation to strain variation is the elastic modulus of concrete, within the range of elastic deformation. The carbonized specimens were tested for the elastic modulus at the carbonation times of 7 d, 14 d, 21 d, and 28 d. Three tests were conducted at each carbonation time and the arithmetic average of the elastic modulus obtained from these tests was considered to be the test value.

## 3. Results and Discussion

**Figure 7:**
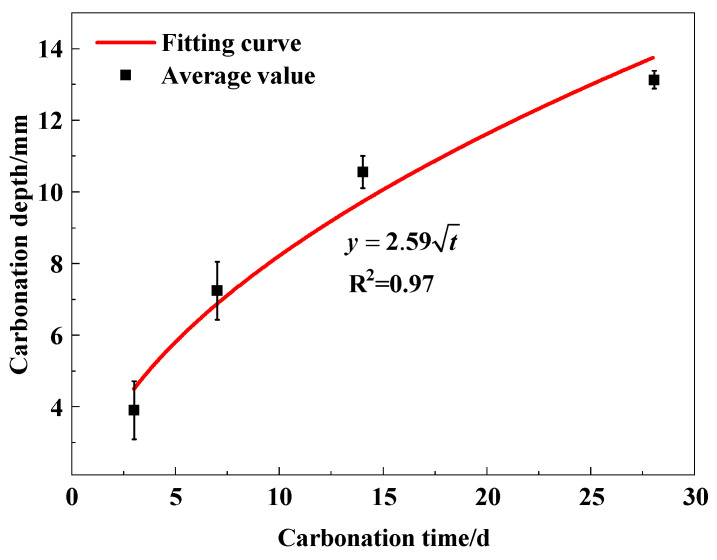
Relationship between carbonation depth and carbonation time.

Table 5 shows the carbonation depth of specimens at different carbonation times. As shown in Figure 7, the carbonation depth increases with carbonization time overall. The growth rate of 3 d~7 d is the fastest, followed by the growth rate of 7 d~14 d, and the growth rate of 14 d~28 d is the slowest. In the early stages of carbonation, the presence of microcracks and pores in the concrete specimen itself, as well as the continuous supply of CO_2_, results in a rapid carbonation reaction and the generation of large amounts of CaCO_3_. As carbonation proceeds, CaCO_3_ precipitation fills the internal pores and microcracks, preventing CO_2_ from entering the interior of the concrete and leading to a decrease in the rate of the carbonation reaction [36]. In previous studies, many scholars have established mathematical models for concrete carbonation based on Fick’s first law of diffusion [12]. The generally accepted general form is shown as Equation (1).

(3) $$y=\alpha \sqrt{t}$$

where *y* is the carbonation depth; *t* is the carbonation time; and α is the carbonation rate coefficient. 

A mathematical model of carbonation depth (*y*) and carbonation time (*t*) is established based on Equation (3), as shown in Figure 7. The correlation coefficient is R^2^ = 0.97. Its fitting carbonation rate coefficient (*α*) is 2.59 in Figure 7.

Furthermore, we give a comparison of carbonation models between our work and the previous work [37], as shown in Table 6. The carbonation rate coefficient is observed to increase with an increase in the water–cement ratio, according to the literature [37]. The fitting carbonation rate coefficient of concrete, with the water–cement ratio of 0.6 in our work, is 2.59, which is less than 3.94 with a water–cement ratio of 0.65 and more than 2.53 with a water–cement ratio of 0.55. Accordingly, the proposed mathematical model here is in good agreement with the existed carbonation model, which confirms the accuracy of both our experimental data and the model itself.

**Figure 8:**
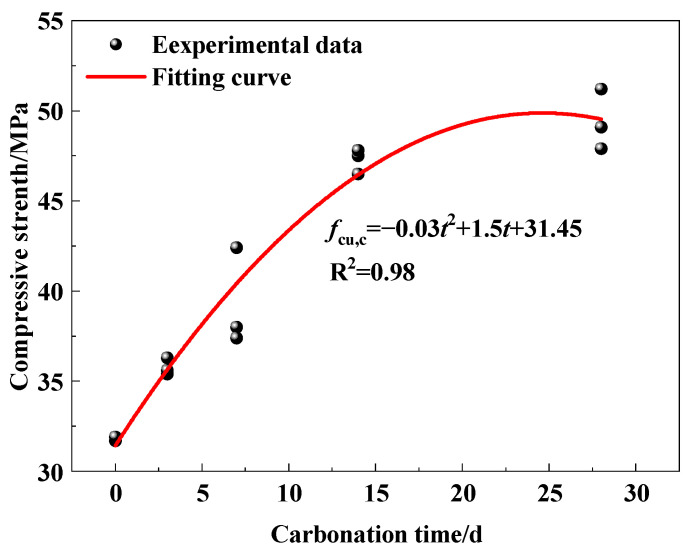
Relationship between compressive strength and carbonation time.

Figure 8 demonstrates the relationship between the compressive strength (*f*_cu,c_) of specimens and carbonation time (*t*). It is fitted as Equation (4). The correlation coefficient is R^2^ = 0.98. Thus, the compressive strength of concrete under carbonation shows a binomial relationship with the carbonation time, which is consistent with previous research [38].

*f*_cu,c_ = −0.03*t*^2^ + 1.5*t* + 31.45 (4)

We observe that the compressive strength increases rapidly from 0 d to 14 d of carbonation time, and grows slowly from 14 d to 28 d. This phenomenon is mainly due to the carbonation reaction that can generate products such as CaCO_3_, which reduces the porosity of concrete [39]. Moreover, with the increase in humidity, water molecules enter the interior of the concrete and continue to hydrate with substances that are not fully hydrated, resulting in a more compact internal structure. At the late stage of carbonation, the concrete specimens become highly compacted, with the internal pores mostly blocked, so the compressive strength does not increase.

**Figure 9:**
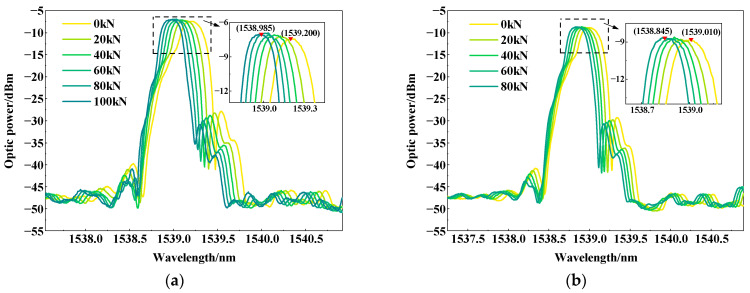
FBG wavelength shifts of carbonized specimens under graded load: (**a**) the carbonation time of 7 d; (**b**) the carbonation time of 14 d.

Figure 9 shows the variation in the wavelength of FBG sensors in concrete specimens with carbonation times of 7 d and 14 d under a stepped load. The wavelength of the reflection peak undergoes a blue shift with the application of step load. In the specimen carbonized for 7 d, the reflection peak wavelength of the FBG sensor is 1539.2 nm when no load is applied. As the load is applied to 100 kN, its wavelength shifts to 1539.985 nm. In the specimen carbonized for 14 d, the wavelengths are 1539.010 nm and 1538.845 nm at 0 kN and 80 kN, respectively. The internal strain of concrete was calculated by the correspondence between the wavelength shift of FBG and its strain. The stress value is the ratio of the applied load value to the area of the cubic specimens. Ultimately, the modulus of elasticity of concrete was derived by dividing the change in stress by the change in strain.

**Figure 10:**
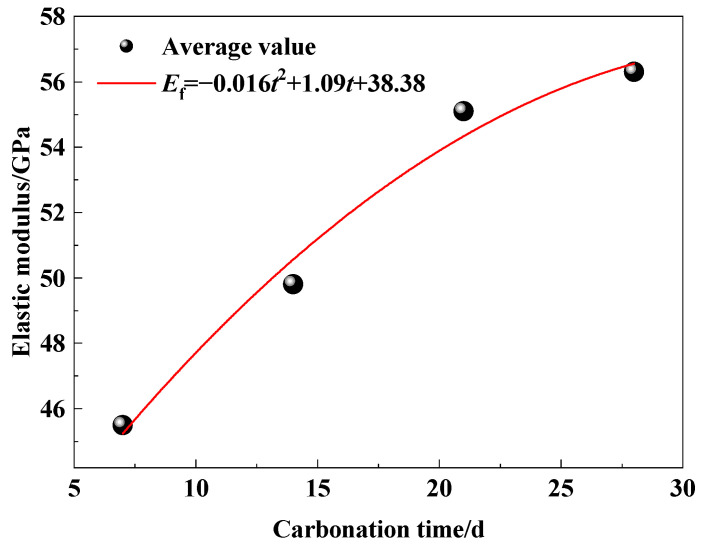
Relationship between elastic modulus measured by FBG and carbonation time.

Figure 10 indicates the relationship between the elastic modulus of concrete, measured by the FBG, and carbonation time. The vertical axis is the elastic modulus, which is the ratio of the variation of stress to that of strain. It is evident that the trend of elastic modulus variation over carbonization time is essentially in line with the trends of compressive strength and carbonation depth of concrete. The elastic modulus increases speedily from 7 d to 21 d, and gradually slows down from 21 d to 28 d. Hence, utilizing FBG to measure the elastic modulus for characterizing the advancement of concrete carbonation is a feasible method. The relationship between the elastic modulus, measured by FBG (*E*_f_), and carbonation time (*t*) is fitted as Equation (5). The correlation coefficient is R^2^ = 0.98.

*E*_f_ = −0.016*t*^2^ + 1.09*t* + 38.38 (5)

It can be seen that the elastic modulus versus carbonation time also exhibits a binomial relationship, which is consistent with the mathematical model of compressive strength with carbonation time. These results agree with the correlation of compressive strength–elastic modulus proposed by Yang [40].

## 4. Conclusions

In this work, the authors explored the effect of carbonation on the mechanical properties of concrete. The elastic modulus under different carbonation times was obtained by embedding the FBG sensor in the concrete. At the same time, mathematical models were established between the three indicators of concrete carbonation depth, compressive strength, and elastic modulus, and carbonation time. The results suggest that FBG can be utilized for assessing concrete carbonation without causing harm to the concrete’s inherent structure. This offers a novel approach for monitoring concrete carbonation in real-time at practical engineering sites. It should be pointed out that this work is currently limited to the laboratory, and further developments should focus on field experiments. The main conclusions are as follows.

(1) Carbonation depth (*y*) increases with carbonation time (*t*), growing rapidly in the early stages of carbonation and leveling off in the later stages. The mathematical model between the two is developed as $y=2.59\sqrt{t}$.

(2) The relationship between compressive strength (*f*_cu,c_) of concrete and carbonation time (*t*) was investigated through compression tests. Initially, the strength of concrete grows rapidly, and, subsequently, it gradually slows down until it remains unchanged for 28 d. Its pattern of change with carbonation time is fitted as *f*_cu,c_ = −0.03*t*^2^ +1.5*t* +31.45.

(3) The variation trend of the elastic modulus of concrete is consistent with the compressive strength, which shows a binomial relationship. The relation between elastic modulus, measured by FBG (*E*_f_), and carbonation time is (*t*) is *E*_f_ = −0.016*t*^2^ + 1.09*t* + 38.38. It is indicated that the elastic modulus of concrete can be characterized by measuring the carbonation of concrete using FBG.

## Figures and Tables

**Table 1:** C30 concrete mix design proportions.

Material	Mix Density (kg/m^3^)
Cement	325
Fine aggregate	714
Coarse aggregate	1166
Water	195

**Table 2:** Performance parameters of T130 cable sensor.

Parameter	Specifications
GFRP cable diameter	1 mm
Strain sensing sensitivity	0.7 pm/με
FBG wavelengths	1526.6\1531.4\1539.2\1539.0\1543.0\1543.1\1546.6\1546.8\1549.6\1551.3\1553.4\1556.3 nm
FBG reflectivity	>75%
FBG reflection BW (FWHM)	0.3 nm
FBG length	10 mm

**Table 3:** Compressive strength of specimens.

Curing Age (d)	Compressive Load (kN)	Compressive Strength (MPa)
3	147.7	14
7	205.7	19.5
28	334.7	31.8

**Table 4:** Conditions of accelerated carbonation test.

Temperature (°C)	Humidity (%)	Concentration of CO_2_ (%)
20 ± 2	70 ± 5	20 ± 3

**Table 5:** Carbonation depth of specimens at different carbonation times.

Carbonation Time (d)	Carbonation Depth (mm)
3	3.9
7	7.3
14	10.6
28	13.1

**Table 6:** Comparison of the concrete carbonation models.

Data Source	Water–Cement Ratio	Carbonation Rate Coefficient	R^2^
Our work	0.60	2.59	0.97
Jin [31]	0.45	1.30	0.84
	0.55	2.53	0.87
	0.65	3.94	0.90

## Data Availability

The data presented in this study are available on request from the corresponding author. The data are not publicly available due to privacy.
